# Personalized Medicine for PLA2R1-Related Membranous Nephropathy: A Multicenter Randomized Control Trial

**DOI:** 10.3389/fmed.2020.00412

**Published:** 2020-08-13

**Authors:** Vesna Brglez, Sonia Boyer-Suavet, Kévin Zorzi, Céline Fernandez, Eric Fontas, Vincent Esnault, Barbara Seitz-Polski

**Affiliations:** ^1^Centre de Référence Maladies Rares Syndrome Néphrotique Idiopathique, Centre Hospitalier Universitaire de Nice, Université Côte d'Azur, Nice, France; ^2^Unité de Recherche Clinique de la Côte d'Azur (UR2CA), Université Côte d'Azur, Nice, France; ^3^Département de Recherche Clinique et Innovation, Centre Hospitalier Universitaire de Nice, Université Côte d'Azur, Nice, France; ^4^Service de Néphrologie-Dialyse-Transplantation, Centre Hospitalier Universitaire de Nice, Université Côte d'Azur, Nice, France; ^5^Laboratoire d'Immunologie, Centre Hospitalier Universitaire de Nice, Université Côte d'Azur, Nice, France

**Keywords:** membranous nephropathy, PLA2R1, epitope spreading, rituximab, personalized treatment, randomized prospective trial

## Abstract

**Background:** Membranous Nephropathy (MN) is a rare autoimmune disease related to PLA2R1 antibodies in 70% of cases. One third of patients enter in spontaneous remission. PLA2R1 epitopes in MN have been characterized in four different domains of PLA2R1 and a mechanism of epitope spreading from the immunodominant CysR domain to CTLD1 and/or CTLD7 and/or CTLD8 domains has been associated with poor prognosis. Epitope spreading could predict spontaneous remission (45% in non-spreader patients vs. 0.05% in spreader patients). The comparison of different regimens of rituximab dosing showed that: (i) early remission rate depends on rituximab dosing, (ii) low dose could be enough for patients with anti-PLA2R1 activity restricted to CysR, (iii) high dose may be required for spreader patients. This study aims to evaluate the efficacy of personalized treatment in PLA2R1-related MN depending on the epitope spreading status, in comparison to the established GEMRITUX protocol.

**Methods:** A multicenter, randomized, controlled, prospective clinical trial will be conducted in 22 French hospitals. Sixty-four consecutive patients with PLA2R1-related MN will be randomly assigned to either the control group following the GEMRITUX protocol (symptomatic treatment for 6 months, if the nephrotic syndrome (NS) persists at month-6, two 375 mg/m^2^ rituximab infusions at 1 week interval) or the personalized treatment group (patients with no epitope spreading at month-0 will be treated with symptomatic treatment for 6 months, if NS persists at month-6, two 375 mg/m^2^ rituximab infusions at 1 week interval; patients with epitope spreading at month-0 or month-6 with persistent NS will be treated immediately with two 1 g rituximab infusions at 2 week interval). The primary study outcome is the rate of clinical remission at month-12. The secondary outcomes include complete and partial remissions, immunological remissions, relapses, proteinuria, albuminuria, serum creatinine, eGFR, PLA2R1 antibody titers, severe infections, lymphocyte counts and lymphocyte phenotype, residual rituximab levels at month-3 and neutralizing anti-rituximab antibodies at month-6 and month-12 after rituximab treatment.

**Discussion:** The results of this trial will confirm whether personalized treatment of PLA2R1-driven nephrotic MN is more efficient to induce clinical remission than the established GEMRITUX protocol, and may thus contribute to improved remission rates and reduced relapse rates.

**Trial registration:** NCT 03804359 trial number. Registered on 15th January 2019.

## Introduction

Membranous nephropathy (MN) is a rare, but severe autoimmune disease with an incidence of 1.3 cases/100,000 people/year in France (1) and a major cause of nephrotic syndrome in adults (2). Membranous nephropathy is defined by subepithelial immune deposits containing IgGs. It can be idiopathic (or primary) without any identified cause (70–80% of cases), or secondary to clinical disorders such as hepatitis B, systematic lupus erythematosus, cancer or drug side effect (3). Spontaneous remission occurs in about one third of patients, and kidney failure in another third (4, 5). Idiopathic MN (iMN) is directed against a podocyte antigen, such as neutral endopeptidase in the neonate, M-type phospholipase A2 receptor (PLA2R1), thrombospondin type-1 domain-containing 7A (THSD7A) and NELL-1 in 70–80, 2–5, and 2–7% of adult patients, respectively (6–9). The pathogenic role of PLA2R1 antibodies (PLA2R1-Ab) has recently been proven *in vitro* (10). Antibody titers usually rise during clinically active phases and decrease before normalization of proteinuria (11, 12). High titers of PLA2R1-Ab at presentation and their persistence predict poor clinical outcome (13, 14).

PLA2R1 is a 180-kDa membrane receptor with a large extracellular region comprising 10 distinct globular domains: a cysteine-rich domain (CysR), a fibronectin type II domain (FNII), and 8 distinct C-type lectin domains (CTLD1-8) (15). Multiple epitopes have been identified within PLA2R1 domain, including an immunodominant epitope in the CysR domain that is targeted by all PLA2R1-positive MN patients (16–18). Three additional C-terminal epitopes exist in CTLD1, CTLD7 and CTLD8 domains, recognized by only a subset of patients (18, 19), and a mechanism of epitope spreading has been proposed from the immunodominant CysR domain to the C-terminal domains (18). Patients with anti-CysR-restricted activity are younger, have lower proteinuria, and exhibit a higher rate of spontaneous remission and a low rate of renal failure progression, while high PLA2R1-Ab activity and epitope spreading to either CTLD1 and/or CTLD7 are independent risk factors of poor renal prognosis (18) and response to treatment (20).

The treatment of iMN is controversial. Kidney Disease Improving Global Outcomes (KDIGO) guidelines recommend a supportive symptomatic treatment with blockers of the renin-angiotensin system and diuretics, and immunosuppressive therapy only in the case of renal function deterioration, complication or persistent nephrotic syndrome (21). Therefore, immunosuppressive treatment is often started only after significant and potentially irreversible complications. On the other hand, an unnecessarily early start of immunosuppression can be futile in patients who develop remission with symptomatic treatment. Therefore, there is a need for better predictors of renal outcome in iMN.

The anti-CD20 antibody rituximab has been increasingly used in iMN. Rituximab can trigger B-cell death and induce depletion of PLA2R1-Ab and clinical remission in 60–80% of patients with iMN in several non-randomized studies (11, 22, 23). Its long-term efficacy was established in a recent randomized controlled trial (GEMRITUX) (24). Seventy-five iMN patients with nephrotic syndrome after 6-months therapy with non-immunosuppressive antiproteinuric treatment (NIAT) were randomly allocated to 375 mg/m^2^ rituximab plus NIAT on days 1 and 8 or NIAT alone. Remission rate was not statistically different between the two groups after 6 months, but it was at last observed time point. The negative results of this study at 6 months raise the question of the selection of patients that received immunosuppressive therapy and of the dose of rituximab used. Indeed, the protocol of rituximab that should be used in heavily nephrotic patients remains controversial (25–27). Pharmacokinetic studies have demonstrated a large inter-individual variability, related either to disease or genetic factors, which could explain differences in clinical response (28–32). In a comparison of two treatment regimens of rituximab in two cohorts of anti-PLA2R1 positive MN patients, early clinical remission was more frequent in the cohort from Nice treated with two 1 g infusions at 2-week interval in comparison to the GEMRITUX cohort treated with two 375 mg/m^2^ infusions at 1-week interval (33). Remission correlated with serum rituximab level and CD19 count at month-3. All patients with antibodies restricted to CysR domain (non-spreaders) entered into remission at last observation regardless of the protocol while patients with epitope spreading had a higher chance of remission with a high dose of rituximab.

Several conclusions could be made: (i) epitope spreading at baseline defined as positivity for either anti-CTLD1 and/or anti-CTLD7 antibodies in serum should be considered for early therapeutic intervention: spontaneous remission occurred in 45% of cases in non-spreader patients vs. 0.05% in spreader patients (20), (ii) early remission rate depends on rituximab dosing, (iii) low dose of rituximab could be enough for patients with anti-PLA2R1 activity restricted to CysR, (iv) high dose of rituximab may be required for patients with epitope spreading.

In this clinical trial, we will compare two therapeutic strategies in patients with PLA2R1-related iMN to induce clinical remission of the nephrotic syndrome. The primary objective of the study is to compare the efficacy of a personalized treatment (stratifying the patients according to their epitope spreading status at month-0 and month-6 and treating them accordingly with either low or high dose rituximab) with the GEMRITUX therapeutic protocol (low dose rituximab after 6 months of symptomatic treatment) to induce clinical remission of the nephrotic syndrome at month-12 in patients with nephrotic iMN driven by anti-PLA2R1 antibodies.

## Methods and Analysis

### Design

This is a two-armed, randomized, open label, multicenter (22 sites), prospective trial. The study will last 6 years: inclusion period will last 4 years with 2 years of follow-up.

### Selection/Treatment of Subjects

#### Conventional Management of iMN Patients

All adult patients with a nephrotic syndrome should have a kidney biopsy and should receive symptomatic treatment. When the diagnosis of MN is confirmed, MN causes are investigated with additional tests comprising CT-scan, serology tests (HBV or with PCR in cases of positivity of antigen HbS or anti-HbC antibody, HBC, HIV), anti-nuclear, anti-PLA2R1 and anti-THSD7A antibodies. After 6 months of NIAT and a persistent nephrotic syndrome, immunosuppressive therapy is started. Follow-up is scheduled every 3 months.

#### Inclusion Criteria

Participants will be included if they fulfill the following conditions: aged 18 years or more, anti-PLA2R1 activity detected by ELISA or Euroimmun IF, nephrotic syndrome defined by proteinuria > 3.5 g/24 h (or UPCR > 3.5 g/g) and serum albumin < 30 g/L at diagnosis, eGFR (CKD-EPI) > 30 ml/min/1,73 m^2^ at diagnosis, symptomatic treatment according to KDIGO guidelines: maximal tolerated dose of NIAT (angiotensin-converting enzyme inhibitor and/or angiotensin 2 receptor blockers, diuretics and statins), medical insurance, provided a signed informed consent, and having understood and accepted the need for long-term medical follow-up. Woman of child-bearing age must be using an effective method of contraception. Inclusion criteria are summarized in Table 1.

**Table 1:** Inclusion and exclusion criteria.

Inclusion criteria:
- Age 18 years or more
- Anti-PLA2R1 activity detected by ELISA or Euroimmun IF
- Nephrotic syndrome defined by proteinuria > 3.5 g/24 h (or UPCR > 3.5 g/g) and serum albumin < 30 g/L at diagnosis
- eGFR (CKD-EPI) > 30 ml/min/1,73 m^2^ at diagnosis
- Symptomatic treatment according to KDIGO guidelines: maximal tolerated dose of NIAT (angiotensin-converting enzyme inhibitor and/or angiotensin 2 receptor blockers, diuretics and statins)
- Medical insurance
- Signed informed consent
- Having understood and accepted the need for long-term medical follow-up
- Woman of child-bearing age must be using an effective method of contraception
Exclusion criteria:
- Secondary MN: MN related to cancer, infections, systemic lupus erythematosus, drugs
- Anti-PLA2R1 antibodies not confirmed by central analysis (in this case the patient will be replaced)
- Pregnancy or breastfeeding
- Immunosuppressive treatment in the last 6 months
- Presence of anti-rituximab antibodies for relapsed patients at inclusion
- Cancer under treatment
- Patients with complicated nephrotic syndrome that would require early immunosuppressive treatment (thrombosis, acute renal failure …)
- Patients with active, severe infections or active hepatitis B
- Hypersensitivity to the active substance or to murine proteins, or to any of the other excipients
- Patients in a severely immunocompromised state
- Severe heart failure (New York Heart Association Class IV) or severe, uncontrolled cardiac disease
- Patients unable to give an informed consent

*CKD-EPI, Chronic Kidney Disease-EPIdemiology collaboration; eGFR, estimated glomerular filtration rate; ELISA, enzyme-linked immunosorbent assay; IF, indirect immunofluorescence; KDIGO, Kidney Disease Improving Global Outcome; MN, membranous nephropathy; NIAT, non-immunosuppressive antiproteinuric treatment; PLA2R1-Ab, antibodies for phospholipase A2 receptor; Rituximab-Ab, antibodies for rituximab; UPCR, urinary protein/creatine ratio*.

#### Exclusion Criteria

Patients with any of the following conditions will be excluded: secondary MN (MN related to cancer, infections, systemic lupus erythematosus, drugs), anti-PLA2R1 antibodies not confirmed by central analysis (in this case the patient will be replaced), pregnancy or breastfeeding, immunosuppressive treatment in the last 6 months, cancer under treatment, complicated nephrotic syndrome that would require early immunosuppressive treatment (thrombosis, acute renal failure), severe infections or active hepatitis B, hypersensitivity to the active substance or to murine proteins or to any of the other excipients, severely immunocompromised state, severe heart failure (New York Heart Association Class IV) or severe uncontrolled cardiac disease, presence of anti-rituximab antibodies in relapsing MN patients (34), unable to give an informed consent. Exclusion criteria are summarized in Table 1.

#### Sample Size

The calculation of remission rate in the personalized arm was based on preliminary data showing that about 30% of patients with iMN have CysR restricted activity at diagnosis (18), and about 50% of them enter in spontaneous remission after 6 months of NIAT and 40% after NIAT + Rituximab at low doses (375 mg/m^2^ D0 and D7) (20). The remaining 70% of patients have additional CTLD1/7 activity, and about 85% of them enter in remission after repeated treatment with high doses of rituximab (33). We therefore expect a remission rate of 80% at M12 in the personalized arm.

The calculation of remission rate in the control arm was based on data from the literature: 10–21% of iMN patients with NS enter into spontaneous remission after 1-year of NIAT (4, 24) and 35% after NIAT + low doses of rituximab (375 mg/m^2^ D0 and D7) (24). We expect a remission rate of 45% in the control arm.

With β = 0.20 and α = 0.05 (two-sided test), the number of patients required is 29 in each group (Nquery Advisor v 7.0, two group Fisher's-exact test). To account for a 10% rate of loss to follow-up (anticipated as minor in this study as patients are intensively followed for this pathology), the global sample size is 64 patients.

#### Recruitment

We can expect an annual eligible number of patients of 1 or 2 in each of the 22 centers, which are in charge of almost all the cases nationally. Since 50% of patients may refuse to participate, we estimate that 4 years are required to recruit the calculated sample size.

Patients with clinical diagnosis of MN will be tested for the presence of PLA2R1-Ab. The selection of the patients fulfilling the inclusion criteria will not delay the start of the symptomatic treatment according to KDIGO guidelines. If the patient meets the criteria for recruitment to the study, they will be informed of the study by the referring nephrologist. Full information will be supplied orally, together with written information.

#### Randomization

After signing the study consent (taken by an appropriately trained physician), participants will be randomized in one of the two treatment arms: (a) GEMRITUX treatment, (b) personalized treatment. Randomization will be balanced (1:1). Centralized block randomization will be conducted by the Department of Clinical Research and Innovation at Nice University Hospital. Due to small study sample and a high number of participating centers, it does not seem appropriate to account for the center as a stratification parameter. Randomization will be integrated in the electronic case report file (e-CRF) devised specifically for the study with Open Clinica® software. Using their personal access details to login, the investigators will provide the necessary patient information (i.e., the first letters of their first and last names and their month and year of birth) for random allocation to treatment by an online randomization module (RedCap®). Randomization can then take place around the clock. The treatment group and inclusion number for the patient will be relayed to the investigator and the central lab team. The patient's trial records will then be created automatically, allowing data to be entered.

#### Blinding

This randomized controlled trial will be an open label study.

### Intervention

#### Investigational Product

The investigational product rituximab will be stored in a secure area according to local regulations. The investigational product will be dispensed only from official study sites by authorized personnel according to local regulations. Biogaran® will provide Rituximab (Truxima®) at no cost for this study. Rituximab will be provided in open-label containers. The labels will contain the protocol prefix, batch number, content, storage conditions, and dispensing instructions along with the Investigational New Drug (IND) caution statement. Investigational product documentation will be maintained, including documentation of drug storage, administration and, as applicable, storage temperatures, reconstitution, and use of required processes (e.g., required diluents, administration sets). Rituximab vials will be stored at a temperature of 2–8°C, protected from light. Care will be taken to assure sterility of the prepared solution as the product does not contain any anti-microbial preservative or bacteriostatic agent. Rituximab will be administered via IV infusion, using a volumetric pump start at 100 mg/h then increase to 400 mg/h. The drug can be diluted with 0.9% normal saline or glucosal 5% for delivery but the total drug concentration of the solution cannot be below 1 mg/ml. Rituximab could be associated with perfalgan 1 g IV, Polaramine 5 mg and Solumedrol 100 mg.

#### Treatment and Follow-Up

All patients will be scheduled at M3, M6, M9, M12, M18, and M24 post-treatment for a routine follow-up, and will benefit from a symptomatic antihypertensive and antiproteinuric treatment throughout the study period according to the KDIGO 2012 guidelines. All patients will be vaccinated (pneumococcal and influenza vaccine) between randomization and first injection at the latest. Rituximab treatment will be scheduled between 5 and 14 days after the visit for the relevant patients.

##### GEMRITUX protocol

The patients from the GEMRITUX group will be treated according to the GEMRITUX protocol. At inclusion, the patients will receive symptomatic antihypertensive and antiproteinuric therapy for 6 months, and they will be reevaluated and stratified at M6. If the patient exhibits an active disease (UPCR remains > 3.5 g/g and albuminemia < 30 g/l), they will receive two 375 mg/m^2^ rituximab infusions at 1-week interval after M6 visit. If the patient is in remission (partial or complete), symptomatic treatment will be continued.

##### Personalized protocol

In the personalized group, patients will be stratified at inclusion/randomization according to their epitope profile. Home-made ELISAs will be used to determine the epitope profile of each patient as described previously (18, 20, 33). Briefly, 96-well microplates will be coated with anti-HA antibody (1:5,000, Sigma-Aldrich) diluted in 20 mM Tris pH 8.0 overnight at 4°C, then blocked with SeramunBlock (Seramun Diagnostica) for 2 h and washed three times with PBS-Tween 0.05%. PLA2R1 antigens expressed as recombinant HA-tagged proteins in the medium of HEK cells, or medium from mock-transfected HEK cells serving as negative control, will be incubated in the wells for 2 h and washed. Patients' sera diluted in 0.1% (m/v) low-fat dry milk in PBS will then be added to wells and incubated for 2 h and washed. Plates will then be incubated for 1 h with anti-human IgG4 horseradish peroxidase (HRP)-conjugated secondary antibody (1:7,500, Southern Biotech) diluted in SeramunStab ST (Seramun Diagnostica) and washed. The signal will be revealed by the addition of tetramethylbenzidine peroxidase substrate (TMB, Interchim) for 15 min before stopping the reaction with 1.2 N HCl, and the plate will be read at 450 nm. The threshold of positivity of each antigen (CysR, CTLD1, and CTLD7) will be determined using a ROC curve, and the value for each individual patient will be corrected for the background value obtained using mock-transfected medium from HEK cells. Appropriate positive and negative controls will be run on each plate, as well as a standard curve for each antigen using predefined dilutions of serum from a highly positive patient allowing the conversion of optical density values into RU/mL using a 5-parameter logistic curve using GraphPad Prism 7. Patients will be considered as non-spreaders if their serum reacts only to the CysR domain, while the patients with an additional signal to either CTLD1 or CTLD7 domains or both will be considered as spreaders.

Patients with anti-CysR-restricted activity at M0 have a spontaneous remission rate from 43 to 50% at M6 under NIAT only (18, 20), and will therefore be treated with symptomatic antihypertensive and antiproteinuric treatment for 6 months. At M6, these patients will be reevaluated for their epitope profile. If the patient is in remission (partial or complete), symptomatic treatment will be continued. In the case they exhibit anti-CysR restricted activity with active disease (UPCR remains > 3.5 g/g and albuminemia < 30 g/l), they have a 70% chance of remission with low dose rituximab (20) and will therefore receive two 375 mg/m^2^ rituximab infusions at 1-week interval after the M6 visit. Patients with additional anti-CTLD1/7 activity at M6 with persisting active disease (UPCR remains > 3.5 g/g and albuminemia < 30 g/l) have 0.05% to 17% chance of spontaneous remission under NIAT (18, 20) and 24% or 64% chance of remission under low or high dose of rituximab, respectively (20), and will therefore be treated with high dose of rituximab (two 1 g rituximab infusions at 2-week interval).

**Figure 1:**
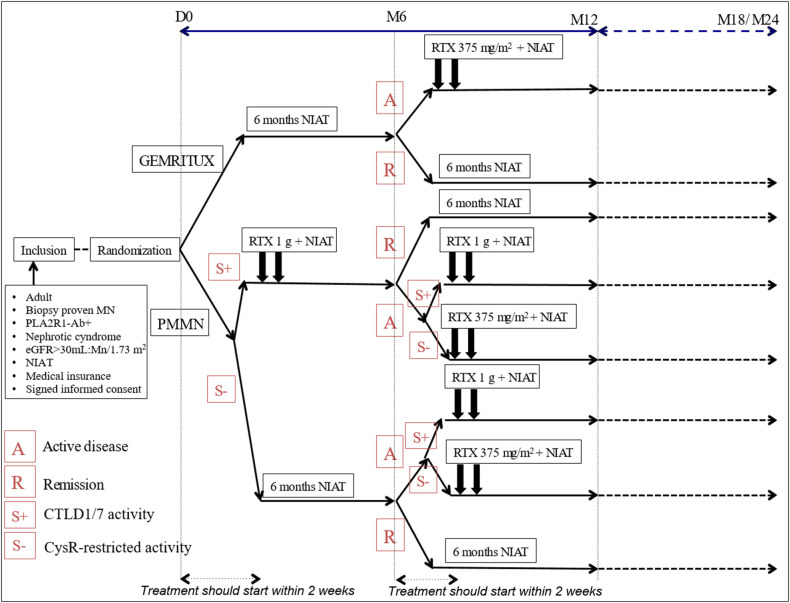
Detailed overview of the PMMN study protocol. A, active disease; eGFR, estimate glomerular filtration rate; MN, membranous nephropathy; NIAT, Non immunosuppressive antiproteinuric treatment; PLA2R1-Ab, antibodies for phospholipase A2 receptor: R, remission; RTX, rituximab; S-, CysR-restricted activity; S+, CTLD1/7 activity.

Patients with additional CTLD1/7 activity at M0 and active disease (UPCR > 3.5 g/g and albuminemia < 30 g/l) will immediately be treated with high dose of rituximab (two 1 g rituximab infusions at 2-week interval), and reevaluated at M6. Symptomatic treatment alone will be continued for the patients achieving partial or complete remission at M6. Among patients with still active disease (UPCR remains > 3.5 g/g and albuminemia < 30 g/l) at M6, those with restricted anti-CysR activity will receive two 375 mg/m^2^ rituximab infusions, while those with additional anti-CTLD1/7 activity will receive two 1 g rituximab infusions at 2-week interval (Figure 1).

#### Patient Data Gathered

Patient data will be collected and directly entered in the patient e-CRF at different time points: inclusion, randomization (D0), month-3 (M3), month-6 (M6), month-9 (M9), month-12 (M12), month-18 (M18), and last follow-up at month-24 (M24). The data collected will be the following: consultation date; demographic data (month and year of birth, gender); history of MN; paraclinic tests to eliminate secondary MN when performed (body scan, anti-nuclear antibodies, HBV, HCV serological tests); current treatment and changes in concomitant treatments; clinical examination (weight, blood pressure, edema); serious adverse events; blood sample (urea, creatinine, electrolytes (Na, K, Cl, HCO3), protein, albumin, full blood count); urine collection on a spot morning sample (protein, albumin, urea, creatinine); centralized assays for both groups (two dry tubes of 5 ml of blood and two EDTA tubes of 2 ml of blood will be shipped to Nice at room temperature for PLA2R1-Ab, epitope profile determination, rituximab serum levels, anti-rituximab antibodies, and IL-35, B and T cells counts) (Table 2).

**Table 2:** Schedule of participant enrollment, interventions and assessments.

	**Study period**							
	**Enrollment**	**Post-allocation (intervention period)**						
	**- 1 week**	**M0**	**M3**	**M6**	**M9**	**M12**	**M18**	**M24**
**Enrollment**								
Eligibility screen	X							
Informed consent	X							
Randomization	X							
**Interventions**								
NIAT								
Rituximab								
1 GEMRITUX protocol								
a) Remission								
b) Active disease				2 × 375 mg/m^2^				
2 Personalized protocol								
a) Remission								
b) Active disease: S-				2 × 375 mg/m^2^				
c) Active disease: S+		2 × 1 g		2 × 1 g				
**Assessments**								
Vaccination		X						
Medical history		X						
Demographics		X						
Physical exam		X	X	X	X	X	X	X
Concomitant medications		X	X	X	X	X	X	X
Serious adverse events		X	X	X	X	X	X	X
eGFR		X	X	X	X	X	X	X
Urine protein/creatinine		X	X	X	X	X	X	X
Urine albumin/creatinine		X	X	X	X	X	X	X
Albuminemia		X	X	X	X	X	X	X
PLA2R1-Ab		X	X	X	X	X	X	X
Epitope profile		X	X	X	X	X	X	X
Residual rituximabemia			X	X	X	X	X	
Rituximab-Ab		X	X	X	X	X	X	
IL-35		X	X	X	X	X	X	X
B and T cells count		X	X	X	X	X	X	X

*eGFR, estimated glomerular filtration rate; IL-35, interleukin-35; PLA2R1-Ab, antibodies for phospholipase A2 receptor; Rituximab-Ab, antibodies for rituximab; S-, CysR-restricted activity, no epitope spreading; S+, CTLD1/7 activity, epitope spreading*.

#### Evaluation Criteria

Clinical remission will be defined as a composite criterion combining (KDIGO definitions): complete clinical remission (urinary protein/creatinine ratio (UPCR) < 0.3 g/g in spot morning urine samples and serum albumin > 35 g/L and eGFR > 60 ml/min/1.73 m^2^), partial clinical remission: UPCR < 3.5 g/g with a decrease > 50% from baseline and serum albumin > 30 g/L and increase of serum creatinine lower than 20% at M12.

Secondary evaluation criteria include: (a) immunological remission: full PLA2R1-Ab depletion measured by ELISA (titer < 14 RU/ml); (b) proteinuria and albuminuria measured on urine sample (proteinuria-to-creatinine ratio or albuminuria-to-creatinine ratio g/g) or collected during 1 day (g per day); (c) change in proteinuria expressed as percentage change in proteinuria from baseline to M6, M9, M12, M18, M24; (d) serum creatinine measured in blood sample in μmol/l and eGFR using the CKD-EPI formula; (e) change in serum creatinine and eGFR expressed as percentage change from baseline to M6, M9, M12, M18, M24; (f) PLA2R1-Ab titer measured by ELISA in RU/ml; (g) severe infections are defined as infections that led to hospitalization during study follow-up; (h) responders are patients who entered in partial or complete remission at M12; (i) relapse is defined by an increased proteinuria > 3.5 g/g after remission at M12.

Patients from both groups will be analyzed jointly. The following risk factors will be studied: (a) lymphocyte counts: B cells (CD19, transitional, mature and memory) and T cells (CD3, CD4, CD8 and TReg) measured in blood sample at D0, M3, M6, M9, M12, M18, M24; (b) serum level of IL-35 (pg/ml) measured in blood sample at D0, M3, M6, M9, M12, M18, M24 using ELISA; (c) residual serum rituximab levels (μg/ml) measured in blood sample 3 months after treatment with rituximab using ELISA; (d) neutralizing anti-rituximab antibodies (ng/ml) measured in blood sample 6 months and 12 months after treatment with rituximab.

#### Sample and Data Collection and Management

Non-fasted blood samples will be shipped on the same day at room temperature to the Laboratory of Immunology in Nice University Hospital, overseen by Dr Seitz-Polski, analyzed and results will be provided to the study site within 48 h. All samples will be retained after analysis (except if consent is withdrawn) to pursue other research goals and will be stored at −80°C in a secured freezer located in the Laboratory of Immunology in Nice University Hospital. No genetic study will be performed on the samples. The Nice University Hospital where the collection is stored will be responsible for declaring it to the Research minister and the Agence Régionale d'Hospitalization. The cell bank has all of the informatics tools and software necessary for conformance with the national bioethical laws. The rules governing sample anonymity will be followed.

In accordance with Good Clinical Practice, at the end of the trial, all documents relating to the protocol will be archived for a period of 15 years by the principal investigator. They will be located in a lockable room providing adequate safeguards against fire, water damage, light, or malice.

A case report file (CRF) will be specifically designed for study data collection. The Clinical Research Assistant responsible for the sponsorship of the study and the Data Manager of the Department of Clinical Research and Innovation at Nice will design the CRF, in coordination with, and under the responsibility of, the principal investigator. The study data will be recorded in the electronic record (e-CRF), implemented by the Data Manager of the Department of Clinical Research and Innovation using Open Clinica® software from the finalized paper CRF. Parameter specification and the implementation of the e-CRF for data collection, including users training, will be the responsibility of the Department of Clinical Research and Innovation. Investigators and clinical research assistants in each center will take responsibility for data collection and for entering it directly into the e-CRF. The data will be securely stored, with specific access rights granted to members of the study team according to their role.

Data quality control will be performed on the e-CRF, using the patient medical file, by the sponsor during the planned monitoring visits by the Department of Clinical Research and Innovation's Clinical Research Officer. Once the final data will have been entered, checks for their validity and coherence will be performed by the Data Manager of the Department of Clinical Research and Innovation and requests for verification issued. Throughout the study any modifications to the database will be recorded, enabling a full audit trail. Finally, the database will be frozen and signed off by the principal investigator, the data manager and the head of biometric department at the Department of Clinical Research and Innovation. No modification of the data will be possible after this time. The frozen database, together with the data management report, will then be transferred to the statistician for analysis.

### Statistical Analyses

#### Analysis Strategy

The statistical analyses will be performed by the biostatistician of the Department of Clinical Research and Innovation at Nice University Hospital. Before each analysis is performed, the conditions for the application of the tests that used will be verified. The various tests will be considered significant at a threshold of 5% (unless otherwise specified). Continuous variables will be described using the number of observations (N), arithmetic mean (Mean), standard deviation, minimum (MIN), median (Median), and maximum (MAX) values. Categorical variables will be summarized by absolute (N) and relative frequencies (%). The statistical analyses will be performed using SAS Enterprise Guide 5.1 software (Copyright (c) 2012 by SAS Institute Inc., Cary, NC, USA). The study patients will be analyzed according to the intention to treat principle (ITT). Each patient will be analyzed as a part of the group to which he or she was assigned at randomization. A per protocol analysis will also be performed, though the results of this analysis cannot be substituted for those of the intention to treat analysis. The “last observation carried forward” (LOCF) strategy will be used to impute the missing data for the principal objective and, when possible, for the secondary objectives. According to the CONSORT 2010 statements, a flow diagram of the progress through the phases (enrollment, allocation, follow-up, and analysis) of this study will be presented. The number of screening failures and reasons for screening failures will be summarized. The characteristics of patients lost to follow-up over the course of the study will be reported.

#### Descriptive Analyses

The statistical analyses will first present a descriptive analysis of the study population and their measured parameters with absolute and relative frequencies (and their 95% confidence intervals) for the categorical variables, and evaluation of means and distributions, medians and inter quartiles for the quantitative variables. As the CONSORT guidelines recommend, the principal characteristics of the patients will be compared between the two groups at inclusion, but no statistical analysis of this will be performed. The comparability of the two arms will be assessed clinically rather than statistically. A flow chart showing the number of eligible patients, the number of patients included, and the number of patients randomized will be presented.

#### Outcomes Analyses

The primary objective of this study is to compare the clinical remission rate at M12 between patients with and without personalized treatment. This will be done using a Chi-square test or Fisher's exact test in case of small sample size. If necessary, a multivariate logistic regression analysis will be performed to assess the relationship between the clinical remission and the treatment group, adjusted for the potential confounding factors.

The secondary objectives consist in comparing several parameters between both treatment groups (GEMRITUX vs. personalized treatment) at different study time points according to each specific objective. The secondary assessment criteria can be grouped into four categories and analyzed as follows: (a) binary criteria (clinical or immunological remission): Chi-square test or Fisher's exact test in case of small sample size; (b) quantitative criteria: student *t*-test or Mann-Whitney rank sum test in case of non-parametric variables; (c) evolution of quantitative criteria (changes from baseline), defined as the difference between the Mx and D0 values, Δvariable = Variable Mx – Variable D0: analysis of covariance, in which the dependent variable will be the Δvariable and the variable of interest the treatment group; (d) severe infections will be described by type and frequency (absolute and relative), and the rate of patients presenting with at least one severe infection during follow-up will be compared between the two groups using a Chi-square test or Fisher's exact test in case of small sample size. Further multivariate analyses will be eventually discussed in case of imbalanced potential confounding factors between the groups.

## Discussion

Current practice in treatment of MN is variable from center to center, both in terms of the treatment choice as well as dosage. As rituximab is the most widely used immunosuppressor in MN patients, it is imperative to prove its efficacy, while limiting its use to the patients that may benefit the most from it. A recent clinical trial GEMRITUX compared low dose rituximab treatment and NIAT alone, but failed to demonstrate the efficacy of rituximab in inducing remission at 6 months (20), while our data showed that a higher dose rituximab may be more effective, especially for patients with epitope spreading (33). In addition, the predictive value of epitope spreading within PLA2R1 remains controversial (19). Our previous work based on a non-controlled open cohort [69 patients (18)], a controlled well-defined cohort [58 patients (20)] and a comparison of two well-defined cohorts [57 patients (33)] demonstrating better prognosis for patients with antibodies raised against the CysR domain only in comparison to patients with multiple antibodies against CTLD1 and/or CTLD7 domains, and that epitope spreading is an independent prognosis factor of MN remission (20). Reinhard et al., using a large but heterogeneous cohort and two different methods for epitope profiling (western blot and ELISA) found that all patients recognized at least two epitopes in the N- and C-terminal parts of PLA2R1 at diagnosis using both methods optimized for the highest sensitivity and serum diluted at 1:25, arguing all patients are spread beyond the CysR domain. However, they also showed in their supplemental data the results obtained using western blot under standard condition with 1:100 serum dilution, demonstrating that spontaneous remission was higher in the group with epitope activity restricted to the CysR domain (remission rate 68% in non-spreader patients vs. 18% in spreader patients, *p* < 0.001) (19). These findings led us to hypothesize that PLA2R1-Ab may be initially raised against the N-terminal CysR domain with pauci-symptomatic iMN disease: patients in the CysR group are younger, probably at the beginning of the disease. A second immune challenge (allergy, infection …) might then induce intramolecular epitope spreading in PLA2R1 toward the C-terminal end (CTLD1, CTLD7 and/or CTLD8 domains) leading to more active disease, which may happen even before the clinical onset of MN (35, 36).

In light of these new findings, epitope spreading and rituximab dosage emerged as two important factors to consider when choosing treatment protocol for patients with PLA2R1-related MN. While it is reasonable to wait 6 months before any immunosuppressive treatment (as suggested by the current KDIGO guidelines) for the patients with CysR-restricted profile who are likely to enter into spontaneous remission, this may unnecessarily reduce the chances of remission for patients with highly active disease and with epitope spreading who are unlikely to enter spontaneous remission and to respond to low dose rituximab.

This study aims to assess the efficacy of personalized treatment of nephrotic MN patients, stratifying them according to their epitope profile, and adjusting accordingly the timing and the dose of rituximab. At the individual level this study should improve the individual management of patients with iMN, and help to better select patients who should benefit from early and aggressive immunosuppressive strategies. Patients in the personalized arm should expect a remission rate around 85% at 12 months. At the collective level, this study should improve the remission rate and decrease the risk of serious adverse events in iMN treatment. Moreover, ancillary studies will help us to understand resistant forms of iMN and will help us propose new immune therapies, such as inducing TReg therapy, memory B-cell targeting or a new generation of humanized anti-CD20 drugs for patients who develop resistance to rituximab.

## Ethics and Dissemination

### Ethics Approval

This study has been approved by the French Ethics Committee, French National Agency for Medicines and Health Products Safety and French National Commission on Informatics and Liberty. The trial will be conducted in compliance with the principles of the Declaration of Helsinki (2000), Good Clinical Practice and in accordance with all applicable regulatory requirements.

### Consent

Informed consent will be obtained from each subject after the study has been fully described to the subject, and the consenting investigator believes that the subject fully understands the study requirements and can make an informed decision.

### Availability of Data and Material

The trial steering committee participated in the design of the protocol, owns the database, and is free to interpret and publish the data. Abstracts or publications using study data will be submitted with the prior agreement of the steering committee president. These abstracts and publications will be prepared by writing groups appointed by the steering committee. Every publication must be reviewed by the steering committee whose approval must be obtained before submission. The study coordinator will be the first author of the principal study; the last author will be the steering committee president. Any PMMN study co-investigator may propose an ancillary study to the steering committee, addressing this request in writing to the steering committee president. These proposals will be reviewed for methodological soundness by the study's biostatistical unit before being evaluated by the steering committee. Specific additional funding must be obtained to finance these studies. Any data necessary for the ancillary study will then be extracted from the database and sent to the responsible investigator. No ancillary study is to be published before publication of the principal study. The order of the list of authorship will be defined by the steering committee in agreement with the responsible investigator of the ancillary study. The responsible investigator for the ancillary study will sign the paper as first author, and the steering committee president will be last author. The study defines a primary objective, secondary objectives, a methodology, and a statistical schema. All of the co-investigators participated in this research leading to its results and conclusions. These conclusions are the result of the efforts of the whole group, and are not simply a juxtaposition of myriad results obtained by many researchers participating in the study. For this reason, the investigators are not at liberty to publish the isolated results from their own institution. If an investigator wishes to propose an ancillary study, whether or not it uses biological specimens, it must be in conformance with this principle. A copy of the publication will be submitted to Nice University Hospital, the study sponsor, who will necessarily be cited. Apart from the coordinating investigators, the list of authorship will be determined pro rata according to the number of patients recruited and analyzed in the publication in question: two co-authors per center having recruited at least 4 patients, and one co-author for each center recruiting between 2 and 4 patients. Requests for the final dataset can be made through the chief investigator.

## Author Contributions

BS-P and VE conceived of the study and initiated the study design. VB, SB-S, CF, and KZ helped with implementation. BS-P, VE, and SB-S provided medical oversight. EF provided statistical expertise. All authors contributed to refinement of the study protocol and approved the final manuscript.

## Conflict of Interest

Biogaran^®^ will provide Rituximab (Truxima^®^) at no cost for this study. Some co-authors are co-inventors on the patents “Methods and kits for monitoring membranous nephropathy” (BS-P), and “Prognosis and monitoring of membranous nephropathy based on the analysis of PLA2R1 epitope profile and spreading” (BS-P and VE). The remaining authors declare that the research was conducted in the absence of any commercial or financial relationships that could be construed as a potential conflict of interest.

## Abbreviations

Ab: antibodies
CD20: Cluster Differentiation 20
CKD-EPI: Chronic Kidney Disease-EPIdemiology collaboration
CTLD: C-type lectin domain
CysR: Cysteine-rich domain
e-CRF: electronic case report file
eGFR: estimate glomerular filtration rate
HBV: Hepatitis B virus
HCV: Hepatitis C virus
ELISA: Enzyme Linked Immunosorbent Assay
IFA: Immunofluorescence Assay
IL-35: Interleukin 35
iMN: idiopathic Membranous Nephropathy
KDIGO: Kidney Disease Improving Global Outcome
LOCF: Last Observation carried forward
MN: Membranous Nephropathy
NEP: Neutral Endopeptidase
NIAT: Non immunosuppressive antiproteinuric treatment
PLA2R1: M-type phospholipase A2 receptor
THSD7A: Thrombospondin type-1 domain containing 7A
TReg: Lymphocyte T regulator
UACR: Urinary albumin/creatine ratio
UPCR: Urinary protein/creatine ratio.
